# Abbé Sicard

**Published:** 1822-10

**Authors:** 


Died lately, at Paris, in his 80th year, the Abbé Sicard, director of the Institution for the Deaf and Dumb. He was born at Touscret in 1742, and was educated at Toulouse; and early in life entered the church, becoming vicar-general of Condour and a canon of Bourdeaux. M. Cicé, archbishop of the diocese, having resolved to establish an asylum for the deaf and dumb, Sicard was dispatched to Paris, to make himself acquainted with the system of the Abbé de l'Epée, whom he afterwards succeeded in 1789. During the Revolution, learning and benevolence proved no protection, and the Abbé Sicard had nearly shared the fate of the unfortunate Lavoisier. He was detained prisoner till the 4th of September, when he was carried before the National Assembly, where he made a speech, which, joined to the interest made in his behalf, seems to have procured his liberation.

After a considerable suspension of his duties, M. Sicard was at length restored to his charge; and the institution, being now supported by M. Chaptal, at that time minister of the interior, began to flourish. In 1800 a press was established, by which means his pupils became initiated in the art of printing: it was here that the works of the Abbé were principally published. One of his first pupils at Bordeaux was Massieu, who followed him to Paris, and whose astonishing acquirements tended, in a remarkable degree, to give celebrity to the system of education adopted by his master.

The school of the Abbé Sicard was an object of great interest and general attraction to all strangers visiting Paris. The readiness which he always showed in explaining his method, and the proofs of cultivated mind and useful acquirements given by his pupils at their public exercises, have been the means of leading to the establishment of similar schools in various parts of Europe. The name of Sicard is associated with the system of De l'Epée, which he greatly improved, and deservedly ranks high among the benefactors of mankind.

---

### METEOROLOGICAL JOURNAL.

